# Remission from nicotine dependence among people with severe mental illness who received help/services for tobacco/nicotine use

**DOI:** 10.1002/mpr.1845

**Published:** 2020-09-18

**Authors:** Hamzah Alghzawi, Alison Trinkoff, Shijun Zhu, Carla Storr

**Affiliations:** ^1^ Department of Family and Community Health, School of Nursing University of Maryland Baltimore Maryland USA; ^2^ Department of Organizational Systems and Adult Health, School of Nursing University of Maryland Baltimore Maryland USA

**Keywords:** nicotine dependence, remission, severe mental illness, smoking

## Abstract

**Objectives:**

A growing body of evidence supports pharmacological interventions to assist smoking cessation in people with severe mental illness (SMI); that is, lifetime major depressive disorder, bipolar disorders, or schizophrenia. Little is known about whether behavioral services are also associated with high probability of remission from nicotine dependence as compared to other types of help/services received (pharmacological, behavioral, or both).

**Methods:**

A sample of 726 American lifetime adult smokers with SMI and a history of nicotine dependence, who received help/services for tobacco/nicotine use, were identified. These data came from a limited public use dataset, the 2012–2013 NESARC‐III. Survival analysis was used to compare the probability of remission from nicotine dependence and the time needed for full remission from nicotine dependence by type of help/services received for tobacco/nicotine use.

**Results:**

Remission was more frequent among those who received behavioral services. In addition, the average time from onset of nicotine dependence until full remission from nicotine dependence was shorter among those who received behavioral services.

**Conclusions:**

The current study suggests a clinical need for behavioral interventions to promote the probability of remission from nicotine dependence among smokers with SMI. Health care providers could play a role in educating and encouraging smokers with SMI to seek and utilize behavioral services.

## 1 INTRODUCTION

People living with mental illness are more likely to smoke compared to the general population (36.1% vs. 21.4%; Centers for Disease Control and Prevention [CDC], 2013; Jamal et al., 2014). They are also more likely to be dependent on tobacco/nicotine. The prevalence of nicotine dependence among smokers with mental illness is 2–4 times higher than that of the general population (Chou et al., 2016; Rüther et al., 2014). Morbidity and mortality rates are 2–2.5 times higher among smokers with severe mental illness (SMI) as compared to smokers in the general population (Blackwell, Lucas, & Clarke, 2014; WHO, 2017). Hence, smoking cessation efforts and programs that enhance remission from nicotine dependence are important for people with SMI.

Several studies have examined factors associated with remission from nicotine dependence in the general and psychiatric populations. These studies have shown being female, older, white, married, and with higher income, higher education, and later onset of tobacco/nicotine use are associated with higher probability of nicotine dependence remission (Goodwin, Pagura, Spiwak, Lemeshow, & Sareen, 2011; Lopez‐Quintero et al., 2011; Peters, Schwartz, Wang, O'Grady, & Blanco, 2014; Segal, Esan, Burns, & Weinberger, 2017). Lopez‐Quintero et al. (2011) reported that for the general population, the average time (in years) from nicotine dependence onset until full remission, differs by racial/ethnic groups. Nicotine dependence remission occurs approximately 24 years after dependence onset among whites, compared to 35 years among blacks and 16 years among Hispanics. Yet, little understanding exists about whether the time from onset of nicotine dependence until full remission from nicotine dependence in people with SMI differs by type of help/services received for tobacco/nicotine use.

Treatment of nicotine dependence is not routinely provided to smokers with SMI (Evins, Cather, & Laffer, 2015; Schroeder & Morris, 2010). This might be partially related to the health care providers' lack of knowledge of effective programs and tobacco/nicotine use treatment strategies for those with SMI (Missen, Brannelly, & Newton‐Howes, 2013; Rae, Pettey, Aubry, & Stol, 2015). However, evidence from clinical trials suggests that pharmacological services alone or combined with behavioral and psychological support is effective for tobacco/nicotine use and tolerated by smokers with SMI (Aldi, Bertoli, Ferraro, Pezzuto, & Cosci, 2018; Anthenelli et al., 2013; Dubrava & Anthenelli, 2018; Hall et al., 2006; Secades‐Villa, González‐Roz, García‐Pérez, & Becoña, 2017; Tidey & Rohsenow, 2009; Tsoi, Porwal, & Webster, 2013; Williams et al., 2012)

Prior evaluations of therapeutic interventions for nicotine dependence in people with SMI recommend pharmacological services to aid smoking cessation (Aldi et al., 2018; Anghelescu, 2009; Annamalai, Singh, & O'Malley, 2015; Cather et al., 2013; Cerimele & Durango, 2012; Evins et al., 2014; Jun, Tian‐liang, Bin, & Xian‐wei, 2009; Rüther et al., 2014; Williams et al., 2012). Recent systematic review and meta‐analyses suggest the effectiveness of behavioral services, such as acupressure and acupuncture combined with counseling, in smoking cessation (Wang et al., 2019; Zulkifly & Amin, 2019). These studies also recommend conducting more research to confirm the effectiveness of behavioral services. In spite of the considerable research on the effectiveness of pharmacological services for smoking cessation and remission from nicotine dependence, little is known about whether using behavioral services alone or in combination with pharmacological services is also associated with nicotine dependence remission compared to pharmacological services alone (Chou et al., 2016; Klinsophon, Thaveeratitham, Sitthipornvorakul, & Janwantanakul, 2017). Despite the high prevalence of nicotine dependence among smokers with SMI, those people have been excluded from most clinical trials of treatments for nicotine dependence (Evins et al., 2015).

Previous research conducted on the general and psychiatric populations has found several factors associated with the effectiveness of and engagement in tobacco/nicotine cessation efforts. Treatment engagement is positively associated with older age (Cupertino et al., 2007; Khara, Okoli, Nagarajan, Aziz, & Hanley, 2015; Richards et al., 2014), higher education (Cupertino et al., 2007), healthcare coverage (Cupertino et al., 2007; Richards et al., 2014), unemployment (Lee, Hayes, McQuaid, & Borrelli, 2010), severity of mental illness‐related symptoms (Lee et al., 2010; MacPherson, Stipelman, Duplinsky, Brown, & Lejuez, 2008; Richards et al., 2014); history of substance use (Richards et al., 2014); general health (Khara et al., 2015); previous smoking treatment use (Travaglini, Li, Brown, & Bennett, 2017); and late onset of smoking (Aschbrenner, Ferron, Mueser, Bartels, & Brunette, 2015; Khara et al., 2015; Lee et al., 2010; Trainor & Leavey, 2016; Travaglini et al., 2017). These other potential factors (e.g., comorbidity with another mental illness) should be taken into consideration when examining and comparing the probability of remission from nicotine dependence among people with SMI by type of help/services received for tobacco/nicotine use.

Our current study extends the work of prior research by estimating the time and probability of full remission from nicotine dependence in people with SMI by type of help/services received for tobacco/nicotine use. This study contributes to the literature by providing data to enhance our understanding about nicotine dependence remission and improve efforts to promote more effective smoking cessation programs aimed at increasing the likelihood of nicotine dependence remission in people with SMI.

To avoid selection biases that might occur in samples recruited from clinical settings, we used a population‐based sample to estimate the time and probability of nicotine dependence remission people with SMI. The specific aims for this study were: (a) to estimate whether the time from nicotine dependence onset until full nicotine dependence remission differs by type of help/services received for tobacco/nicotine use (pharmacological, behavioral, and both); (b) to estimate differences in the probability of nicotine dependence remission by type of help/services received, controlling for sociodemographic characteristics, comorbidity with another mental illness and smoking‐related factors. We hypothesized that the estimated time from onset of nicotine dependence until full remission and the probability of nicotine dependence remission differs by type of help/services received for tobacco/nicotine use.

## 2 METHODS

### 2.1 Study design, data source, and study sample

A secondary data analysis used a public limited dataset obtained from the 2012–2013 National Epidemiologic Survey on Alcohol and Related Conditions (NESARC‐III) conducted by the National Institute on Alcohol Abuse and Alcoholism (NIAAA). Details on NESARC‐III are published elsewhere (Grant et al., 2014). Briefly, the national sample consisted of 36,309 noninstitutionalized U.S. adults from 18 to 99 years old. Participants were selected through multistage probability sampling, and data were obtained through face‐to‐face and computer assisted interviews, with an overall response rate of 60.1%. Sample weights were developed to adjust for sampling biases and to ensure representativeness of the U.S. population. The Census Bureau and the U.S. Office of Management and Budget confirmed the ethics protocol for this survey. All participants provided informed consent, and they received $90.00 for their participation.

The study sample included people with a history of SMI (major depressive (disorder [75.5%], bipolar disorders [14.1%], or schizophrenia [10.4%]) who met criteria for nicotine dependence with a history of receiving help/services for tobacco/nicotine use. SMIs were assessed using the Alcohol Use Disorder and Associated Disabilities Interview Schedule‐5 (AUDADIS‐5) that has been shown to have concordance with clinical evaluations (*κ* = 0.24–0.59; Hasin et al., 2015). A variable created by the NESARC study team was used to indicate if a participant had lifetime major depressive or bipolar disorder. Lifetime schizophrenia was identified through an item asking, “Did a doctor or other health professional ever tell you that you had schizophrenia or a psychotic illness or episode?” We combined these items into a single variable for lifetime SMI (yes or no).

**FIGURE 1:**
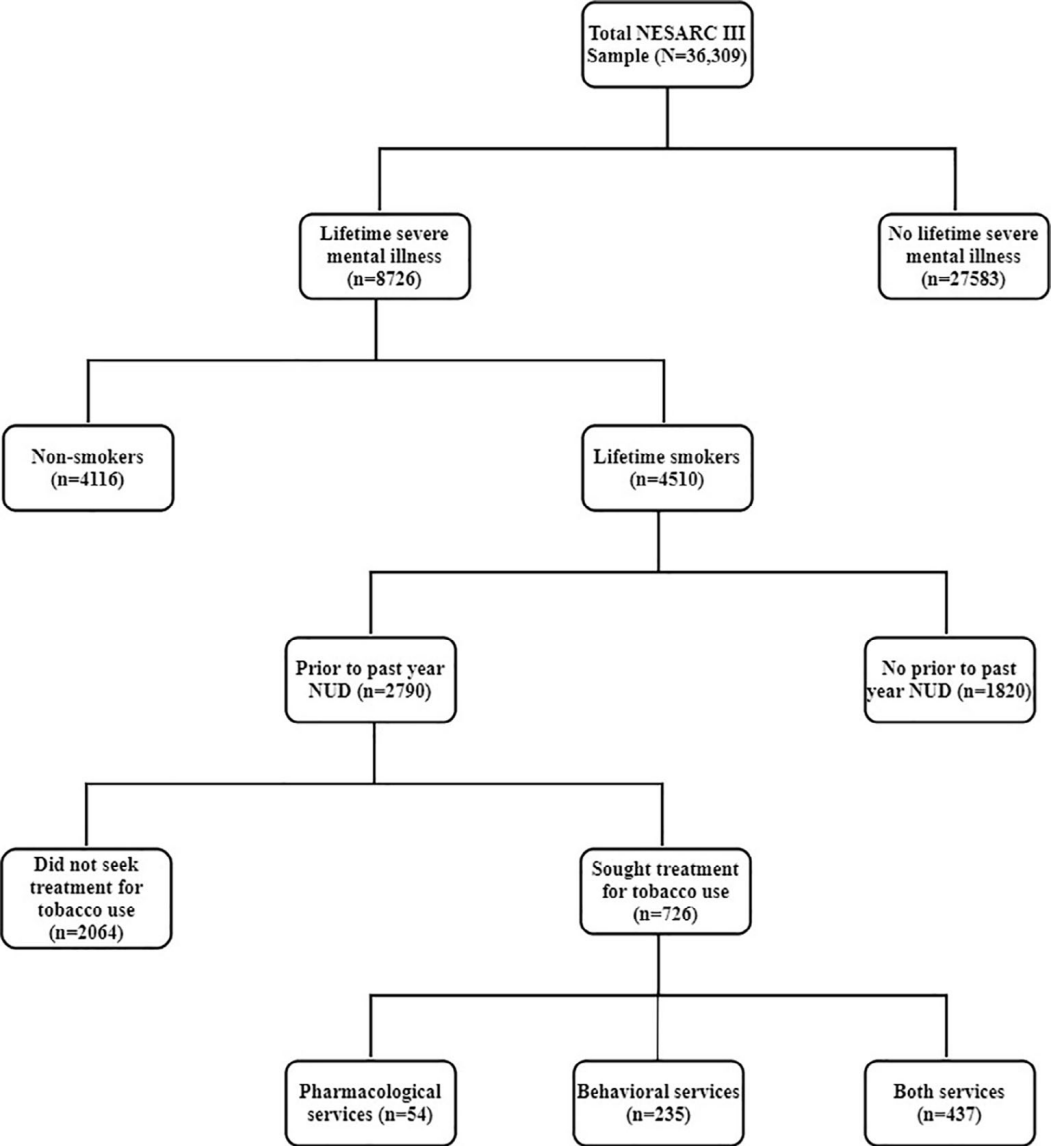
Consort diagram

The AUDADIS‐5 also contained a module that assessed tobacco/nicotine use (Grant et al., 2015). This module included items capturing nicotine dependence criteria with follow‐up questions to determine if it occurred before or within the past 12 months (with nicotine dependence defined as meeting more than 2 of the 11 criteria in a single year). Reliability of the DSM‐5 nicotine dependence (k = 0.50–0.87, intraclass correlation coefficient [ICC] = 0.83–0.84) has been found to be fair to excellent (Grant et al., 2015). A single item assessed if the participant had ever seen anyone for help related to tobacco/nicotine use. Figure 1 is a consort diagram illustrating the sample selection for this study's final sample (*n* = 726).

### 2.2 Measures

#### 2.2.1 Remission

Nicotine dependence remission was assessed using a variable indicating whether or not the participant had a current nicotine dependence (past year nicotine dependence). Individuals in the past year, who did not meet any DSM 5 criteria other than craving, also were classified as having remitted.

#### 2.2.2 Help/services received for tobacco/nicotine use

This was measured by asking if participants had ever gone (in their entire lifetime) anywhere or seen anyone to get help that was in any way to their use of tobacco or nicotine, or if they did anything else to help them quit or cut down on tobacco or nicotine use. Those responding “yes” were then asked a series of questions listing types of assistance received. Study participants were categorized into three groups based on the type of help/services they received for tobacco/nicotine use (pharmacological only, behavioral only, or both). Those who responded “yes” to any of the following were classified as obtaining pharmacological service: “use nicotine patches, lozenges, or gum,” or “have a health professional prescribe any medicine or drug,” whereas those who responded “yes” to any of the following were classified as obtaining behavioral services: “receive acupuncture, acupressure, e‐therapy or meditate,” “go to counseling, family services, or other social services,” “go to a support group,” or “use any other methods to help you quit or cut down.” Those who received both pharmacological and behavioral services were classified into a third group named “both pharmacological/behavioral treatments.”

#### 2.2.3 Tobacco/nicotine use‐related factors

Nicotine use‐related factors that were included in this study are self‐reported age at tobacco/nicotine first use (“the youngest age reported to questions on age when first used cigarettes, cigars, pipe, or snuff”), age at onset of daily smoking (“age started using tobacco/nicotine every day”), type of tobacco/nicotine product used (e.g., cigarettes, cigars, pipe, and snuff/chewing tobacco), quantity of smoking (“usual quantity when used tobacco/nicotine every day [number of times used tobacco/nicotine every day]”), duration of daily smoking in years (“duration [years] when used tobacco/nicotine every day”), and age at tobacco/nicotine last use (“age when last used tobacco/nicotine”).

#### 2.2.4 Mental and substance use comorbid illnesses

Comorbidity with lifetime DSM‐5 mental and substance use comorbid illnesses was also included. The mental comorbid illnesses included dysthymia (persistent chronic depression), any anxiety disorder (e.g., panic, agoraphobia, social phobia, specific phobia, and generalized anxiety), posttraumatic stress disorder, and any personality disorder (e.g., schizotypal, borderline, antisocial). Substance use disorders included alcohol use disorder and any drug use disorder (e.g., sedative/tranquilizer, cannabis, stimulant, cocaine, heroin, hallucination, and inhalant use disorders). Various AUDADIS‐5 modules were used to identify participants meeting criteria for mental and substance use comorbid disorders. The psychometric properties of the modules that were used to identify those disorders ranged from fair to excellent (*κ* = 0.36–0.87, ICCs >0.68; Chou et al., 2016; Grant et al., 2015; Hasin et al., 2015).

#### 2.2.5 Sociodemographic characteristics

Sociodemographic characteristics included in this study are self‐reported age (18–29 years, 30–44 years, 45–64 years, or 65+ years), gender (male vs. female), race‐ethnicity (Hispanic, non‐Hispanic Black, and non‐Hispanic others), education (less than high school, high school, and some college or higher), personal income ($0–19,999; $20,000–$34,000; $35,000–$69,999; $70,000+), marital status (married/living with someone, never married, divorced/separated/ widowed), urbanity (urban vs. rural), region (North, Midwest, South, and West), and general health status (good to excellent vs. poor to fair). Individuals reporting Hispanic or Latino ethnicity were classified as Hispanic regardless of race. Individuals reporting Native American (4.2%) or Asian/Pacific Islander (0.9%), or White (84.4%) were classified as non‐Hispanic others.

### 2.3 Data analysis

Statistical analysis was conducted using SPSS version 25, taking into account the complex sampling design of the NESARC. Weight and stratification variables were applied to ensure that the data were representative of the U.S. population. Descriptive statistics were used to describe all variables by type of help/services received for tobacco/nicotine use. Means and standard deviations were computed for continuous variables, and absolute and relative frequencies were calculated for categorical variables. All variables were compared by type of help/services received for tobacco/nicotine use, using ANOVA‐tests for continuous variables and Chi‐square tests for categorical variables. The pattern of missing data was checked across all variables, and missing data ranged from 0 to 0.4%. Missingness was randomly distributed with no monotonicity indications, and the results of Little's missing completely at random (MCAR) test were not statistically significant (Chi‐square = 6.762, *df* = 6, *p* = .347). Hence, we concluded that missing data were probably MCAR, and missingness was ignorable.

Survival analysis was conducted to estimate the time and probability of nicotine dependence remission by type of help/services received, controlling for other potential covariates and confounders. The outcome of interest was “remission from nicotine dependence.” The difference in age at full nicotine dependence remission minus the age at onset of nicotine dependence defined the length of time participants had nicotine dependence. Participants with nicotine dependence without remission were defined as censored and censoring time was their age at interview minus age at nicotine dependence onset.

The Kaplan–Meier (KM) method was used to obtain univariate descriptive statistics, including the median time from age at onset of nicotine dependence until age at full nicotine dependence remission by type of help/services received. The log‐rank test was used to examine whether the length of time of having nicotine dependence differed by type of help/services received. Then a Cox proportional hazard model was used to analyze the association between the services received and the probability of nicotine dependence remission, controlling for potential confounders and covariates. The Cox proportional hazard model assumptions were checked including linearity of the continuous independent variables, multicollinearity, and proportionality of hazards. The value of the hazard ratio indicated the probability of remission from nicotine dependence with each additional year having nicotine dependence.

## 3 RESULTS

### 3.1 Sample description

The study sample was primarily female (61.1%), non‐Hispanic White (84.0%), urban (75.0%), and from the south (31.2%). Half (51.8%) of the sample was between 45 and 65 years of age, 59.5% had at least some college and 54.1% were married or living with someone. Just above two‐third (69.1%) had personal income below $35,000 and two‐thirds reported they were in good to excellent health (65.1%). Participant characteristics, such as age (*χ* ^2^ = 16.8, *p* < .001), race‐ethnicity (*χ* ^2^ = 16.5, *p* < .01), education (*χ* ^2^ = 8.8, *p* < .01), and personal income (*χ* ^2^ = 17.1, *p* < .05) differed across the type of help/services received (Table 1).

**TABLE 1:** Characteristics of individuals diagnosed with severe mental illness and lifetime nicotine use disorder by type of help/services received for tobacco/nicotine use (NESARC‐III, *N* = 663)

Characteristics	Pharmacological (*n* = 440)^a^	Behavioral (*n* = 38)^b^	Both services (*n* = 185)	*χ* ^2^‐test
	%^c^	%^c^	%^c^	
Gender				
Male Female	37.9 62.1	40.0 60.0	40.9 29.1	0.5
Age				
18–29 years 30–44 years 45–64 years 65+ years	6.4 35.3 51.4 6.9	13.9 9.9 55.2 13.7	9.1 29.0 52.3 9.6	16.8***
Race‐ethnicity				
Non‐Hispanic White Non‐Hispanic Black Hispanic	90.9 4.3 4.8	75.2 11.1 13.7	88.6 5.7 5.7	16.5*
Marital status				
Married/living with someone Divorced/separated/widowed Never married	56.4 30.8 12.8	41.2 43.1 15.7	50.9 38.0 11.1	5.3
Education				
Less than high school High school Some college or higher	2.6 41.0 56.4	2.5 46.1 51.4	1.3 30.6 68.1	8.8**
Personal income				
$0–19,999 $20,000–$34,000 $35,000–$69,999 $70,000+	26.5 43.6 23.6 6.3	20.6 58.8 14.5 6.1	17.2 50.1 20.4 12.3	17.1*
Urbanity				
Urban Rural	76.0 24.0	66.4 33.6	74.0 26.0	1.5
Region				
Northeast Midwest South West	21.6 27.7 30.5 20.2	17.3 16.6 38.5 27.7	23.6 21.9 31.6 22.9	4.9
General health				
Good to excellent Poor to fair	64.5 35.5	63.3 36.7	66.9 33.1	3.1

^a^ Pharmacological services include nicotine patches, lozenges, or gum o any prescribed medicine or drug.

^b^ Behavioral services include: counseling, family services, or other social services; support group; acupuncture, acupressure, e‐therapy or meditation; or any other methods.

^c^ Weighted percentages.

* *p* < .05.

** *p* < .01.

*** *p* < .001.

The proportion with some college was higher among those who received both types of services for tobacco/nicotine use; whereas the proportion between 45 and 65 years of age was higher among those who received behavioral services. Personal income under $35,000 was also reported more often by those receiving behavioral services.

### 3.2 Mental comorbid illnesses

Comorbid mental illnesses were common in the sample. Table 2 compares comorbid disorders by type of help/services received for tobacco/nicotine use. Only comorbidity with a substance use disorder differed across the type of help/services received for tobacco/nicotine use (*χ* ^2^ = 1.9, *p* < .05). Comorbidity with a substance use disorder was more common among those who only received behavioral services (81.8%) than those receiving pharmacological or both services.

**TABLE 2:** Remission from nicotine dependence by mental comorbid illnesses, and smoking‐related factors among individuals with a history of lifetime severe mental illness who received help/services for tobacco/nicotine use (NESARC‐III, *N* = 663)

Variable	Pharmacological^a^ (*n* = 440)	Behavioral^b^ (*n* = 38)	Both services (*n* = 185)	*χ* ^2^‐test
	%^c^	%^c^	%^c^	
Status of nicotine dependence in the past year				
Remitted Persistent	21.8 78.2	43.7 56.3	18.8 81.2	9.3*
Type of severe mental illness				
Schizophrenia Major depressive disorder Bipolar disorder	11.9 68.3 19.8	10.0 76.6 13.4	10.4 75.8 13.8	1.3
Alcohol use disorder Any drug use disorder Any substance use disorder	65.5 37.5 71.8	76.9 57.8 81.8	66.2 33.4 69.4	1.9*
Dysthymia (persistent chronic depression)	20.3	40.5	26.3	8.2
Panic disorder Agoraphobia Social phobia Specific phobia Generalized anxiety disorder Any anxiety disorder Posttraumatic stress disorder	25.7 12.3 18.8 14.7 30.4 59.1 25.2	15.4 11.1 24.4 20.6 17.9 66.5 43.7	29.4 8.1 13.5 17.4 26.7 62.8 35.2	1.2 9.5
Schizotypal personality disorder Borderline personality disorder Antisocial personality disorder Any personality disorder	27.1 43.8 15.6 52.4	13.9 40.8 18.8 49.8	32.2 45.3 23.6 55.3	0.6
Type tobacco product used				7.3
Cigarettes Others^d^ Both	56.6 1.7 41.7	59.4 3.0 37.5	47.4 0.7 52.0	
	Mean (*SE*)^e^	Mean (*SE*)^e^	Mean (*SE*)^e^	ANOVA‐test
Age at first tobacco use	15.1 (0.003)	15.7 (0.013)	14.6 (0.004)	0.4*
Age at onset of daily smoking	17.0 (0.003)	17.6 (0.013)	17.1 (0.004)	0.3
Duration of daily smoking (years)	20.6 (0.007)	15.8 (0.011)	20.5 (0.006)	1.7**
Quantity of daily smoking^f^	19.4 (0.007)	17.5 (0.010)	19.6 (0.006)	0.3
Age at smoking cessation	45.1 (0.016)	44.6 (0.033)	46.5 (0.010)	2.6*

^a^ Pharmacological services include nicotine patches, lozenges, or gum o any prescribed medicine or drug.

^b^ Behavioral services include: counseling, family services, or other social services; support group; acupuncture, acupressure, e‐therapy or meditation; or any other methods.

^c^ Weighted percentages.

^d^ Others tobacco/nicotine products include cigars, pipe, snuff/chewing tobacco, or e‐cigarettes or e‐liquid.

^e^ Weighted means.

^f^ Number of times used tobacco/nicotine every day.

* *p* < .05.

** *p* < .01.

### 3.3 Smoking‐related factors

Factors, such as age at first tobacco/nicotine use (*F* = 0.4, *p* < .05), duration of daily smoking (*F* = 1.7, *p* < .01), and age at smoking cessation (*F* = 2.6, *p* < .01) also differed across the type of help/services received for tobacco/nicotine use. Those who received both services reported earlier age at first tobacco/nicotine use and older age at smoking cessation compared to those who received pharmacological and behavioral services. On the other hand, those who received behavioral reported less duration of daily smoking compared to those who only received pharmacological or both services (see Table 2).

### 3.4 Remission from nicotine dependence by type of help/services

Essentially, out of people with SMI who remitted from nicotine dependence (*n* = 828), only 19.1% had received help/services for tobacco/nicotine use. Out of those who received help/services (*n* = 663), 21.7% had remitted from nicotine dependence. Remission from nicotine dependence differed by type of help/services received for tobacco/nicotine use (*χ* ^2^ = 9.3, *p* < .05). The proportion that remitted with a history of receiving pharmacological services was 17.6%. Remission was more frequent among those who received behavioral services (28.5%) or when both types of services were received (19.6%).

**FIGURE 2:**
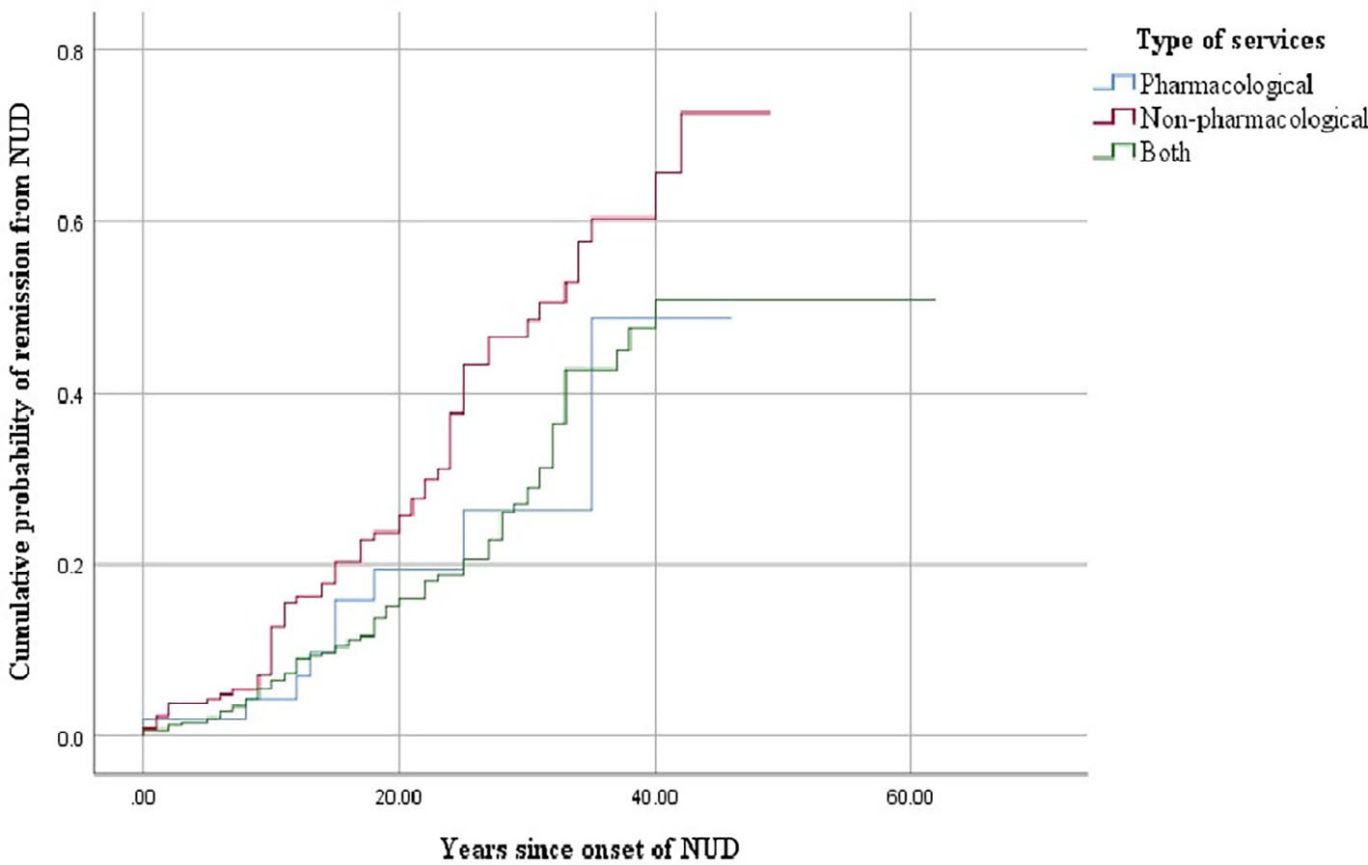
Probability of remission from nicotine dependence over time by type of help/service received for tobacco/nicotine use

The cumulative probability of nicotine dependence remission differed by type of help/services received (log rank = 7.8, *p* < .05). The Kaplan–Meier plot examined the probability of remission from nicotine dependence over time (see Figure 2). The curve of those who received behavioral services only was higher than for those who received pharmacological services or those who received both, indicating that those who received behavioral services had the highest probability of nicotine dependence remission. The average time from onset of nicotine dependence until full nicotine dependence remission among those who received behavioral services was 35 years (95% CI: 32.2, 37.6), compared to 37 years (95% CI: 32.1, 42.3) among those who received pharmacological services and 47 years (95% CI: 43.9, 49.8) for those who received both.

The associations among type of help/services received and the probability of nicotine dependence remission were initially tested using univariate Cox proportional hazard models (see Table 3). Then the potential associations between type of help/services and the probability of remission from nicotine dependence was tested using three multivariate models adjusted for sociodemographic characteristics. Model (1) included SMI type and comorbidity with another mental illness; Model (2) contained smoking‐related factors; and Model (3) combined Model 1 and 2 covariates. All potential correlates reaching statistical significance at 0.15 level in the univariate models were included in the third (final) multivariate model.

**TABLE 3:** Cox proportional hazards model of the association between type of help/services received for tobacco/nicotine use and the probability of remission from nicotine use disorder (NESARC‐III, *N* = 663)

Covariate	Crude	Model 1^a^	Model 2^b^	Model 3^c^
	HR (95% CI)	HR (95% CI)	HR (95% CI)	HR (95% CI)
Type of help/services used				
Pharmacological^d^ Behavioral^e^ Both	1.00 1.33 (1.31, 1.34) 0.67 (0.66, 0.68)	1.00 1.06 (1.05, 1.07) 0.47 (0.46, 0.48)	1.00 1.96 (1.94, 1.98) 1.57 (1.55, 1.58)	1.00 1.95 (1.93, 1.96) 1.52 (1.51, 1.55)
Type of severe mental illness				
Schizophrenia Major depressive disorder Bipolar disorder	1.00 2.84 (2.82, 2.87) 2.45 (2.42, 2.48)	1.00 1.99 (1.97, 2.01) 1.50 (1.48, 1.52)	–	1.00 1.62 (1.60, 1.64) 1.46 (1.45, 1.48)
Substance use disorder				
No Yes	1.00 1.81 (1.79, 1.82)	1.00 1.97 (1.96, 1.98)	–	1.00 0.94 (0.93, 0.95)
Dysthymia				
No Yes	1.00 0 .74 (0.73, 0.75)	1.00 0.77 (0.76, 0.77)	–	1.00 1.06 (1.04, 1.07)
Any anxiety disorder				
No Yes	1.00 1.04 (1.03, 1.05)	1.00 0.99 (0.98, 1.01)	–	1.00 1.08 (1.07, 1.09)
Posttraumatic stress disorder				
No Yes	1.00 0.89 (0.88, 0.90)	1.00 0.96 (0.95, 0.97)	–	1.00 0.83 (0.82, 0.84)
Any personality disorder				
No Yes	1.00 0.78 (0.77, 0.79)	1.00 0.82 (0.81, 0.83)	–	1.00 1.09 (1.08, 1.10)
Type tobacco/nicotine product used	1.00	–	1.00	1.00
Cigarettes Other^f^ Both	0.45 (0.44, 0.46) 0.43 (0.42, 0.44)	–	0.64 (0.63, 0.65) 1.15 (1.14, 1.16)	0.68 (0.67, 0.70) 1.14 (1.13, 1.15)
Age at first tobacco use	1.03 (1.02, 1.04)	–	0.97 (0.96, 0.98)	0.97 (0.96, 0.98)
Age at onset of daily smoking	1.03 (1.02, 1.04)	–	1.08 (1.07, 1.09)	1.09 (1.08, 1.10)
Duration of daily smoking (year)	0.96 (0.95, 0.97)	–	0.99 (0.98, 1.00)	0.99 (0.98, 1.00)
Quantity of daily smoking^g^	1.01 (0.99, 1.02)	–	1.00 (0.99, 1.01)	1.00 (0.99, 1.01)
Age at smoking cessation	0.92 (0.91, 0.93)	–	0.93 (0.92, 0.94)	0.93 (0.92, 0.94)

Abbreviations: CI, confidence interval; HR, hazard ratio; SMI, severe mental illness.

^a^ Adjusted for sociodemographic characteristics, type of SMI, and comorbidity with another mental illness.

^b^ Adjusted for sociodemographic characteristics and smoking‐related factors.

^c^ Adjusted for sociodemographic characteristics, type of SMI, comorbidity with another mental illness, and smoking‐related factors.

^d^ Pharmacological services include nicotine patches, lozenges, or gum o any prescribed medicine or drug.

^e^ Behavioral services include: counseling, family services, or other social services; support group; acupuncture, acupressure, e‐therapy or meditation; or any other methods.

^f^ Others include cigars, pipe, snuff/chewing tobacco, or e‐cigarettes or e‐liquid.

^g^ Number of times used tobacco/nicotine every day.

Table 3 shows the results of the association between each type of help/services received for tobacco/nicotine use and the probability of remission from nicotine dependence. This association was consistent for Models 2 and 3, and differed for Model 1. In Model 1, the probability of nicotine dependence remission was higher among those who received one type of service compared to those who received both, whereas the probability of nicotine dependence remission in Models 2 and 3 was higher among those who received behavioral or both services compared to those who received pharmacological services. The inferiority of combined services in the crude model and Model 1 suggests that smoking factors might have influenced the delivery of combined services. In Model 3, the value of hazard ratio (HR = 1.95, 95% CI: 1.93, 1.97) for those who received behavioral services means that for each additional year of nicotine dependence, the probability of nicotine dependence remission was 1.95 times that of those who received pharmacological services, after adjustment for other potential covariates and confounders. Additionally, the findings showed how SMI type, smoking‐related factors, comorbidity with other mental illnesses were significantly associated with the probability of nicotine dependence remission (see Table 3).

## 4 DISCUSSION

The current study sought to estimate the time and probability of remission from nicotine dependence by type of help/services received for tobacco/nicotine use in people with SMI. We observed that those who received behavioral services had a higher probability of remission from nicotine dependence compared to those who received pharmacological or both services. Interestingly, the probability of remission from nicotine dependence among those who only received behavioral services was higher than those who received both. The finding in some of our analyses that combined services were associated with a lower likelihood of remission suggests that smoking factors and the severity of nicotine dependence might influence the delivery of combined services.

Our findings were consistent with previous studies that found an association between receiving services for tobacco/nicotine use and nicotine dependence remission (Aldi et al., 2018; Annamalai et al., 2015; Rüther et al., 2014; Stubbs, Vancampfort, Bobes, De Hert, & Mitchell, 2015). In addition, the current study extends the prior research with population‐based data.

Our study findings differed from studies that found pharmacological services were more helpful in people with SMI compared to behavioral services (Aldi et al., 2018; Secades‐Villa et al., 2017). However, our findings were consistent with research that has found a combination of pharmacological services and psychoeducation is more effective than pharmacological services alone (at least 1 year; Evins et al., 2015; Klinsophon et al., 2017; Maglione et al., 2017).

Our study suggests that smokers with SMI would benefit from receiving behavioral services for tobacco/nicotine use to improve the probability of nicotine dependence remission. Hence, smokers with SMI should be encouraged to seek and use behavioral services to enhance their odds of nicotine dependence remission. According to Evins et al. (2014), receiving pharmacological services for at least 1 year has shown promise for long‐term abstinence among smokers with SMI. Our study is also congruent with the recent U.S. Public Health Service guidelines for smoking cessation in the general population that recommend receiving a combination of pharmacological services and counseling for smoking cessation as it is more effective than received pharmacological services alone (Maglione et al., 2017).

The study findings should be interpreted while considering common limitations in most studies based on data from a national survey. First, information on nicotine dependence and remission from nicotine dependence was based on self‐report measures and not confirmed by objective measures. Second, data on smoking related factors and receiving services for tobacco/nicotine use might be subject to recall bias and cognitive impairment associated with mental illness. The help/service categories are crudely assessed and additional information on the timing (when attempt made, sequencing of services, etc.) and how often quit attempts were made would provide greater insight into the service utilization patterns of those attempting to quit.

Third, NESARC survey excludes institutionalized populations, who might have a higher probability of having nicotine dependence. Fourth, differential service delivery was not considered in this study due to data limitation. Fifth, the data for this study were collected 6–7 years ago, and therefore might not reflect current practices. Nonetheless, our study used a comprehensive definition of behavioral services by including psychoeducation or counseling, along with family or other social services; support groups; acupuncture, acupressure, e‐therapy or meditation; and any other methods. In addition, study strengths include the large sample size allowing for subgroups analysis, representativeness and relative generalizability, and the careful assessment methods.

## 5 CONCLUSION AND IMPLICATIONS

The current study suggests a clinical need for behavioral interventions to promote the probability of nicotine dependence remission among smokers with SMI. Health care providers could educate and encourage smokers with SMI to seek and use behavioral services for tobacco/nicotine use to improve the probability of their remission from nicotine dependence and facilitate prolonged abstinence. Future research should take into account differential service delivery to examine associations between receiving behavioral services and remission from nicotine dependence. In addition, it would be beneficial to compare the effectiveness of different behavioral services and evaluate them for smokers with SMI.

## DECLARATION OF INTEREST STATEMENT

No conflicts of interest were declared by any author.

## DISCLAIMER

The view and opinions expressed in this report are those of the authors and should not be constructed to represent the views of any of the sponsoring organization or agencies or the U.S. government.

## Data Availability

Data access information for the NESARC‐III is available at https://www.niaaa.nih.gov/research/nesarc‐iii/nesarc‐iii‐data‐access.
